# Response to upadacitinib monotherapy in a patient with eczematid-like pigmented purpuric dermatosis

**DOI:** 10.1016/j.jdcr.2024.10.008

**Published:** 2024-10-30

**Authors:** Aditi Chokshi, Anna Agaronov, Uros Rakita, Penelope Hirt, Francisco Kerdel

**Affiliations:** a Nova Southeastern University Dr. Kiran C. Patel College of Osteopathic Medicine, Davie, Florida; b Department of Dermatology, Larkin Community Hospital, South Miami, Florida; c Florida Academic Dermatology Center, Coral Gables, Florida

**Keywords:** JAK inhibitor, pigmented purpuric dermatosis, upadacitinib

## Introduction

Pigmented purpuric dermatoses (PPDs) are a group of chronic relapsing cutaneous diseases histologically characterized by lymphocytic capillaritis. Five morphologic variants are described with the unifying clinical feature of clusters of petechiae on a background of golden-brown pigmentation.^1^ Eczematid-like purpura of Doucas and Kapetanakis is a rare inflammatory variant of PPD presenting with pruritic and scaly purpuric lesions.^1^ While often idiopathic, venous hypertension, exercise, infections, medications, and gravitational dependence are all contributing factors to PPD pathogenesis.^1^

Universally effective treatments are lacking. Corticosteroids, phototherapy, flavonoids, vitamin C, dapsone, pentoxifylline, and other immunomodulatory agents are used with varying success.^2^ We present a case of a 56-year-old female with refractory eczematid-like purpura of Doucas and Kapetanakis that was successfully treated with upadacitinib.

## Case report

**Fig 1:**
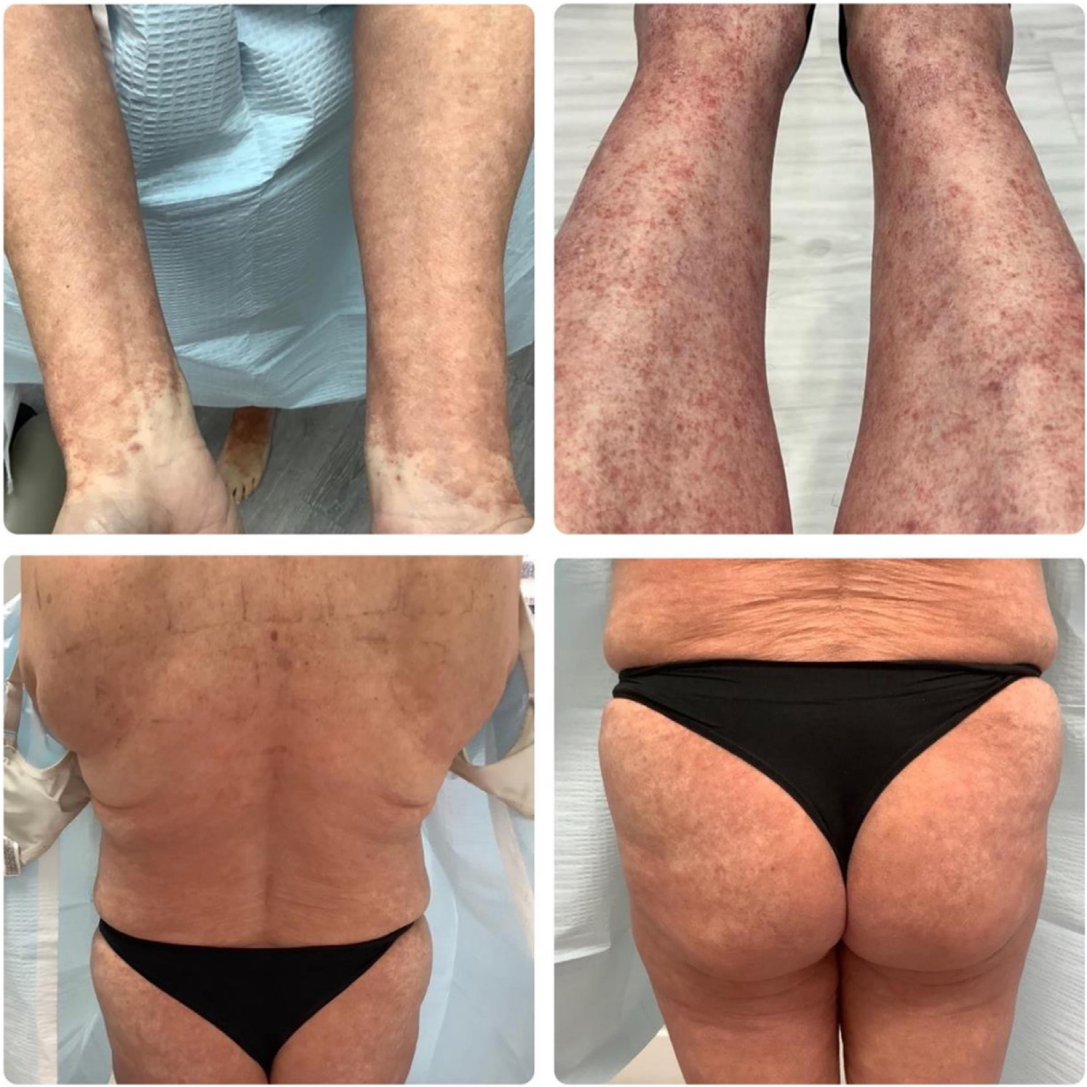
Diffuse eczematous plaques with scattered pinpoint cayenne *pepper*-colored petechial macules.

**Fig 2:**
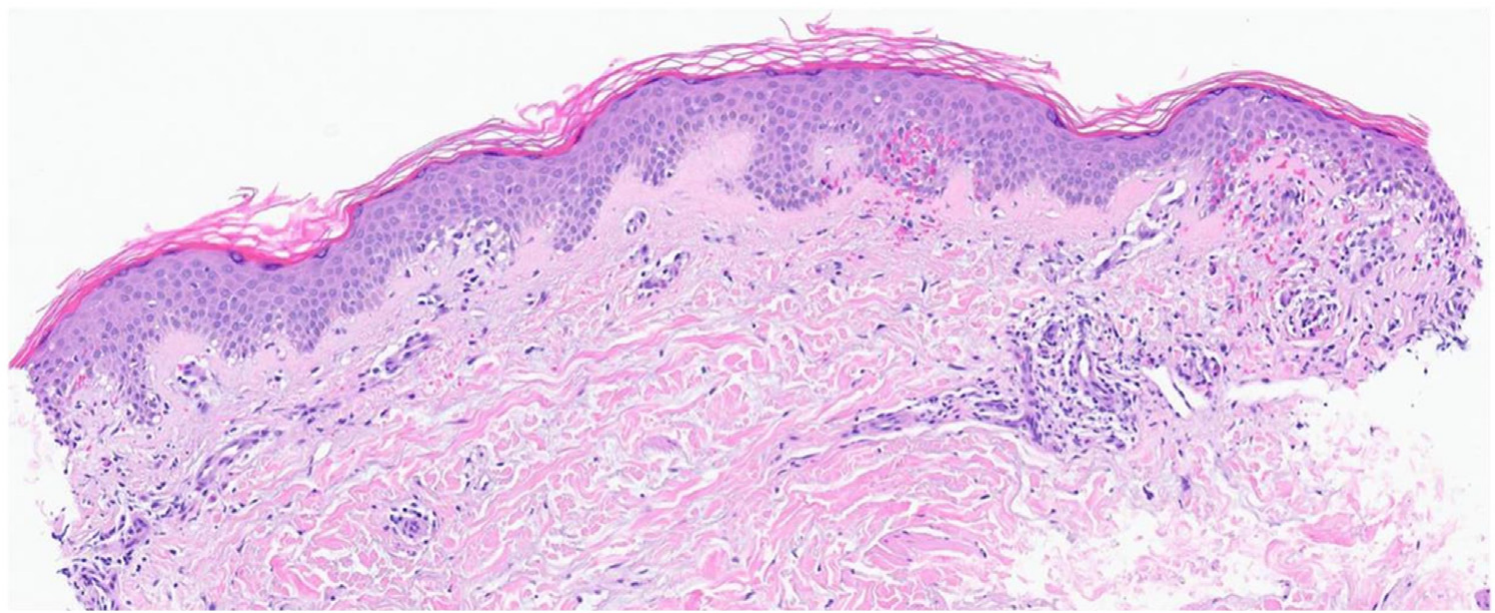
Mild epidermal spongiosis, perivascular lymphocytic infiltrate with extravasated erythrocytes, and rare pigmented macrophages consistent with pigmented purpuric dermatosis.

**Fig 3:**
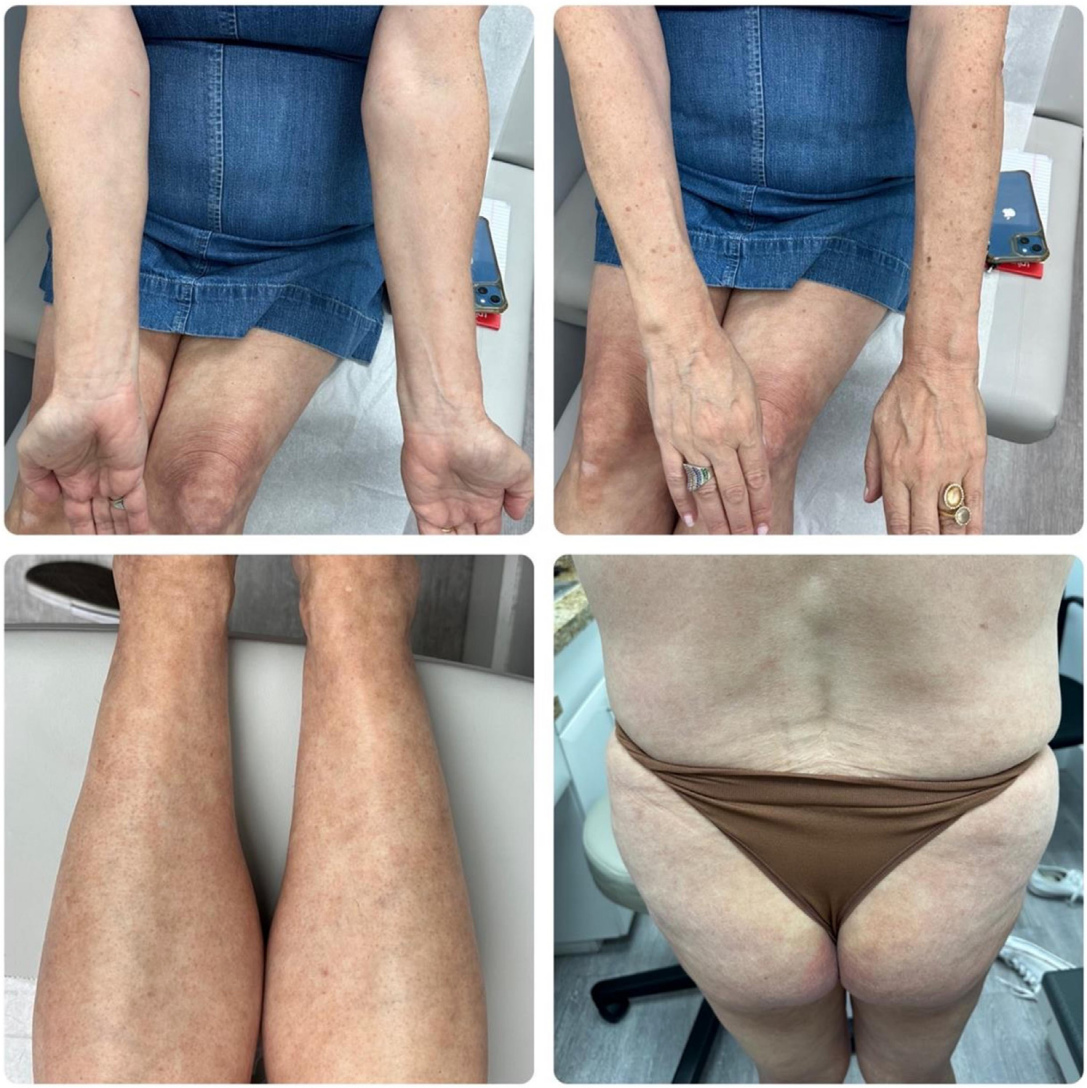
Complete resolution after 2 weeks of upadacitinib monotherapy.

**Fig 4:**
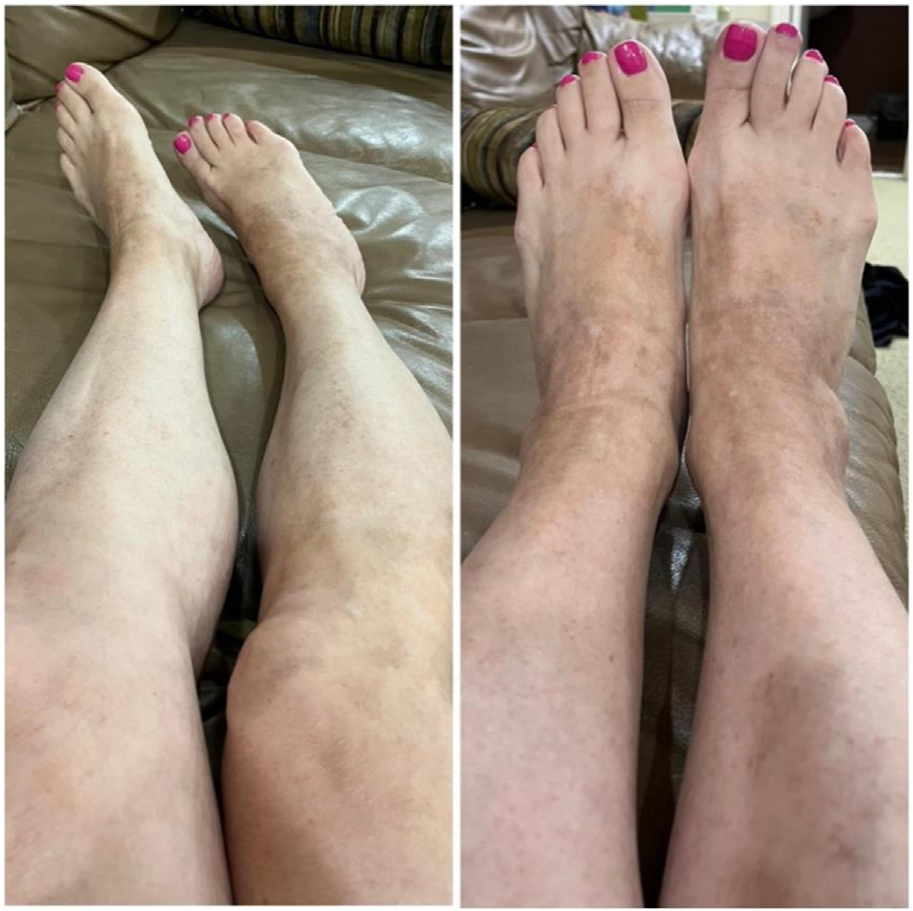
Residual postinflammatory hyperpigmentation 3 months after discontinuation of upadacitinib.

A 56-year-old female presented with a 1.5-year history of pruritic scaly, eczematous, confluent red-pink patches and plaques with scattered petechiae distributed on the bilateral lower and upper extremities, back, and abdomen (Fig 1). Biopsy showed mild epidermal spongiosis, perivascular lymphocytic infiltrate with extravasated erythrocytes, and rare pigmented macrophages consistent with pigmented purpuric dermatosis (Fig 2). She previously failed a 2-month course of topical triamcinolone, topical tacrolimus, and oral pentoxifylline. All prior treatments were discontinued, and she was started on upadacitinib 15 mg daily monotherapy. At the 2-week follow-up appointment, complete resolution of lesions and pruritus was achieved (Fig 3). Upadacitinib was discontinued after 2 additional months of treatment. At the 5-month follow-up since presentation, the patient remains asymptomatic with few localized postinflammatory hyperpigmentation changes with no treatment-related adverse events (Fig 4).

## Discussion

Upregulation of cell adhesion molecules in both endothelial cells and leukocytes is likely the primary immunopathogenic event in all PPDs.^1^ This results in a cell-mediated immunologic response primarily localized to dermal capillaries.^1^ Our report suggests that this pathogenic pathway is at least partially dependent on the Janus kinase (JAK)-signal transducer and activator of transcription signaling cascade.

JAK inhibitors are a family of 4 intracellular tyrosine kinases (JAK1, JAK2, JAK3, and tyrosine kinase 2 proteins) that mediate the signaling of numerous type I and type II cytokines in a variety of cell types and tissues through downstream activation of signal transducer and activator of transcription.^3^ Upadacitinib is a selective oral JAK1 inhibitor approved for a variety of conditions including atopic dermatitis and psoriatic arthritis.^4^ Only 2 prior cases of PPDs managed with JAK1 inhibitors have been published.^5^ These patients had the most common form of PPD, Schamberg disease, for a 1-6 month duration prior to starting JAK1 inhibitor therapy.^5^ They responded to upadacitinib or abrocitinib within 1-3 weeks.^5^ Our patient similarly responded rapidly to upadacitinib, despite suffering for over 1.5 years with a more inflammatory and diffusely distributed PPD variant.

JAKs are increasingly recognized as appropriate therapeutic options in many dermatologic conditions, demonstrating their broad immunomodulatory effects. Our report adds evidence supporting the use of JAK1 inhibitors for PPD management. Additionally, we demonstrate that upadacitinib may be effective for multiple PPD variants, even in long-standing cases. Further research is needed to evaluate the long-term safety and efficacy of JAK inhibitors in the treatment of PPDs.

## Conflicts of interest

None disclosed.

## References

[1] Spigariolo C.B., Giacalone S., Nazzaro G. (2021). Pigmented purpuric dermatoses: a complete narrative review. J Clin Med.

[2] Kimak A., Żebrowska A. (2024). Therapeutic approach in pigmented purpuric dermatoses-A scoping review. Int J Mol Sci.

[3] Chapman S., Kwa M., Gold L.S., Lim H.W. (2022). Janus kinase inhibitors in dermatology: part I. A comprehensive review. J Am Acad Dermatol.

[4] Muddebihal A., Khurana A., Sardana K. (2023). JAK inhibitors in dermatology: the road travelled and path ahead, a narrative review. Expert Rev Clin Pharmacol.

[5] Cao S., Liu Y., Chen S. (2024). JAK1 inhibitor: a promising option for patients with pigmented purpuric dermatoses. J Eur Acad Dermatol Venereol.

